# OsMTP11 is localised at the Golgi and contributes to Mn tolerance

**DOI:** 10.1038/s41598-017-15324-6

**Published:** 2017-11-10

**Authors:** Emily C. Farthing, Paloma K. Menguer, Janette Palma Fett, Lorraine E. Williams

**Affiliations:** 1 Biological Sciences, University of Southampton, Life Sciences Building 85, Highfield Campus, Southampton, SO17 1BJ Hampshire, United Kingdom; 2 Departamento de Botânica, Instituto de Biociências, Universidade Federal do Rio Grande do Sul, Av. Bento Gonçalves 9500, Porto Alegre, 91501-970 Brazil; 3 Centro de Biotecnologia, Universidade Federal do Rio Grande do Sul, Caixa Postal 15005, Porto Alegre, 91501-970 Brazil

## Abstract

Membrane transporters play a key role in obtaining sufficient quantities of manganese (Mn) but also in protecting against Mn toxicity. We have characterized OsMTP11, a member of the Cation Diffusion Facilitator/Metal Tolerance Protein (CDF/MTP) family of metal cation transporters in *Oryza sativa*. We demonstrate that OsMTP11 functions in alleviating Mn toxicity as its expression can rescue the Mn-sensitive phenotype of the Arabidopsis *mtp11-3* knockout mutant. When expressed stably in Arabidopsis and transiently in rice and tobacco, it localises to the Golgi. OsMTP11 partially rescues the Mn-hypersensitivity of the *pmr1* yeast mutant but only slightly alleviates the Zn sensitivity of the *zrc1 cot1* yeast mutant. Overall, these results suggest that OsMTP11 predominantly functions as a Mn-transporting CDF with lower affinity for Zn. Site-directed mutagenesis studies revealed four substitutions in OsMTP11 that appear to alter its transport activity. OsMTP11 harbouring a substitution of leucine 150 to a serine fully rescued *pmr1* Mn-sensitivity at all concentrations tested. The other substitutions, including those at conserved DxxxD domains, reduced complementation of *pmr1* to different levels. This indicates their importance for OsMTP11 function and is a starting point for refining transporter activity/specificity.

## Introduction

Manganese (Mn) is an essential micronutrient in plants. For example, Mn is a vital cofactor in photosystem II, for the water-splitting and oxygen evolution reactions of photosynthesis^1–3^. Mn also plays an important role in detoxification of reactive oxygen species (ROS) via Mn-superoxide dismutase (Mn-SOD), a major antioxidant enzymatic system that scavenges superoxide radicals to convert into H_2_O_2_^4^. However, Mn toxicity becomes a problem when too much Mn is available, with a reduction in plant growth and biomass reported in a range of species under Mn toxicity^5–7^. Other symptoms associated with Mn toxicity include localised chlorosis^8,9^ with necrotic brown spots containing accumulations of oxidized Mn compounds^10,11^. Mn toxicity has also been attributed to a reduction in net photosynthesis and carboxylation efficiency^12–14^. Thus, symptoms of Mn toxicity may lead to losses in agricultural yield. Mn toxicity represents an important problem in tropical, acidic soils, and under conditions which favour a reducing environment, such as water-logged soils with low redox potential^15^.

Plants vary widely in their tolerance to Mn extremes. Interestingly, certain rice cultivars, which are typically grown in flooded or water-logged soils, possess very high toxicity tolerance thresholds; compared to less-tolerant barley cultivars, they are able to accumulate over 30x the amount of Mn in their aerial tissues without displaying toxicity symptoms^16^. To avoid symptoms of both Mn deficiency and toxicity, plants possess various mechanisms to regulate their intracellular Mn concentrations. Membrane transporters play a key role in alleviating both extremes, obtaining sufficient levels of Mn for essential processes and removing Mn from the cytoplasm when accumulating to detrimental levels^17,18^.

The Cation Diffusion Facilitators (CDFs) comprise a ubiquitous superfamily of transporters involved in alleviating heavy metal toxicity across all kingdoms. Also referred to as Metal Tolerance Proteins (MTPs) in plants, the CDFs cluster phylogenetically into clades based on their proposed substrate specificity^19–21^. Some plant members of the Group 8 and 9 MTPs, including MTP8, MTP9, MTP10 and MTP11, have been functionally characterised as Mn transporters; they provide different pathways to alleviate Mn toxicity, such as efflux from the plasma membrane^22–24^ or sequestration into the vacuole^25–28^ or Golgi^29^.

Rice possesses two Group 8 MTPs (OsMTP8 and OsMTP8.1) and three Group 9 MTPs (OsMTP9, OsMTP11 and OsMTP11.1), which may contribute to the high tolerance of rice to Mn toxicity^23,26^. Two members have been characterised in detail to date: OsMTP9 functions at the plasma membrane for efflux of Mn into the root stele to aid root-to-shoot translocation^23^, while OsMTP8.1 localises to the tonoplast in shoots serving to sequester Mn in the vacuole^26^. OsMTP11 and OsMTP11.1 are the focus of this present study. OsMTP11 fused to green fluorescent protein (GFP) has recently been reported to localise to the entire onion epidermal cell cytoplasm, with the authors using prediction programs to hypothesise even distribution at the plasma membrane and endomembrane systems^30^. Colocalisation markers were not employed and therefore the membrane localisation of OsMTP11 warrants further investigation. AtMTP11, the homologous Group 9 member from *Arabidopsis thaliana*, has been localised in two independent studies. In one, it was proposed to function by sequestering Mn into the pre-vacuolar compartment (PVC) for subsequent storage in the vacuole^31^, while in another it was localised to the Golgi, serving in vesicular trafficking to the plasma membrane for exocytosis from the cell^32^. The Golgi-based hypothesis is corroborated by the increased accumulation of Mn in leaves of Arabidopsis *mtp11* mutants, which are also hypersensitive to elevated Mn. Expression of AtMTP11 in metal-sensitive yeast mutants conferred tolerance to elevated Mn (and to a lesser extent Cu), but no increased tolerance was detected with a range of other metals (Zn, Co or Ni)^32^.

All putative MTPs and CDFs characterised to date possess the CDF signature sequence across transmembrane domains (TMDs) 2 and 3 and the interconnecting loop. Additionally, a DxxxD motif is found on TMDs 2 and 5 of all characterised plant Mn-MTPs, which is substituted for HxxxD in Zn-MTPs; this motif is also conserved in non-plant CDFs, but the first aspartate (D) is not always strictly conserved^19^. Mutational studies have shown this motif to be important for function in Mn-transporting OsMTP8.1^33^ and Zn-transporting OsMTP1^34^, and also in non-plant Mn-transporting CDFs, including the human SLC30A10^35,36^ and MntE from *Streptococcus pneumoniae* ^37^.

The aim of this study was to isolate and characterise OsMTP11 using two heterologous systems: Arabidopsis, analysing the capacity of OsMTP11 to rescue the high-Mn susceptibility of the Arabidopsis *mtp11* mutant; and *Saccharomyces cerevisiae*, testing the ability of OsMTP11 to complement yeast mutants defective in metal transport. The latter system was also used for analysing the importance of key residues in OsMTP11 for function. Additionally, we used stable expression in Arabidopsis and transient expression in rice and tobacco to more precisely determine the cellular localisation of OsMTP11.

## Results

### Sequence analysis of OsMTP11

**Figure 1:**
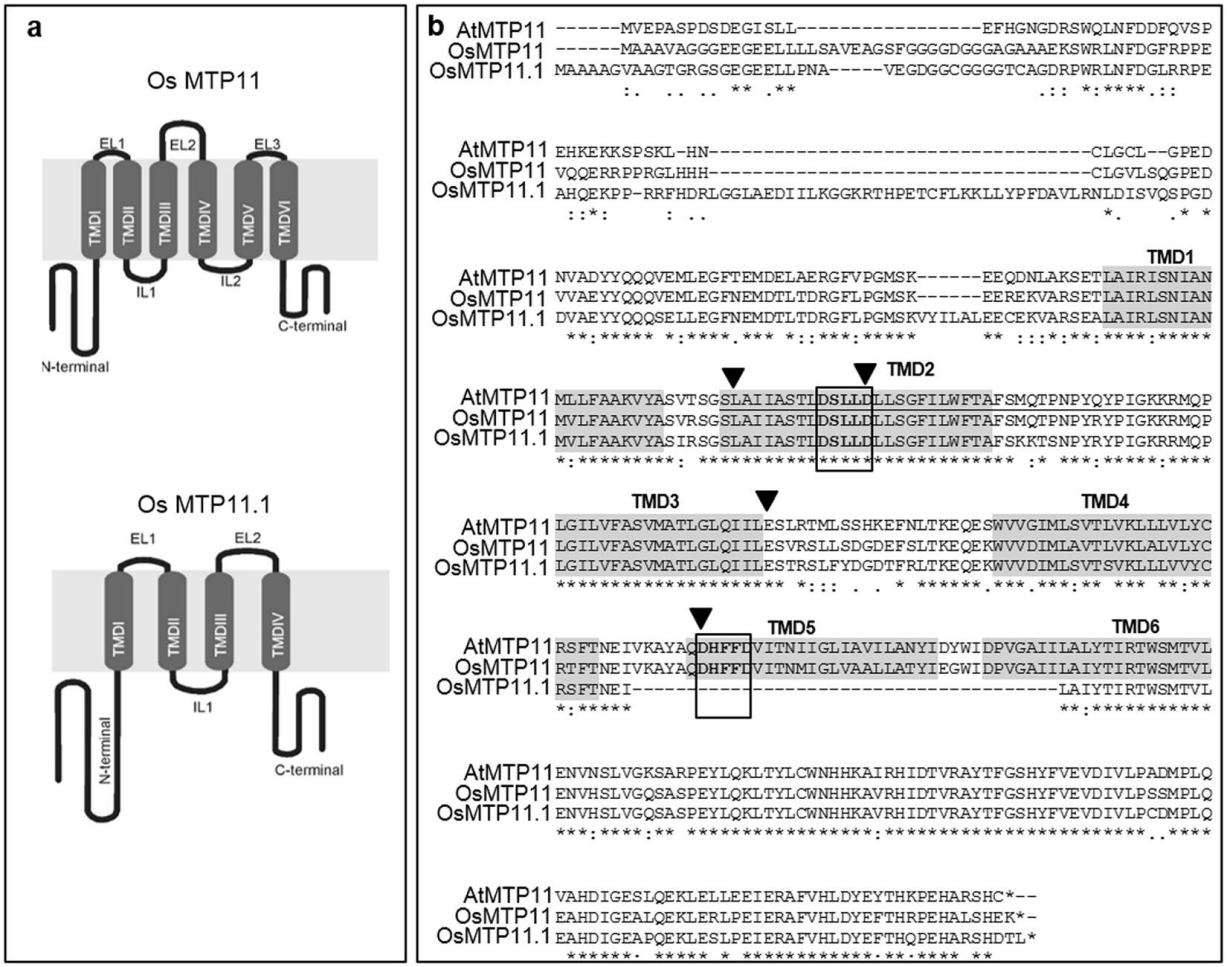
Sequence analysis of OsMTP11 and OsMTP11.1. (**a**) hypothetical membrane topology of OsMTP11 and OsMTP11.1 predicted according to the Phobius program^66^. EL, extracytosolic loop; IL, intracytosolic loop. (**b**) Alignment of OsMTP11, OsMTP11.1 and At MTP11 using ClustalOmega. Transmembrane domains (TMDs) labelled according to predictions from Phobius. Underlined, CDF signature sequence; boxed, DxxxD domains on TMDs 2 and 5 characteristic of Mn-MTPs^19^. Arrows mark the residues of OsMTP11 that were mutated in this study: L150, D162, E213 and D267.

The study of MTPs in rice is important to elucidate mechanisms involved in metal homeostasis in crops. Searching the *Oryza sativa* genome confirmed the presence of both OsMTP11 and OsMTP11.1 in Group 9, together with OsMTP9, but no additional MTP10 was identified. The full-length cDNA fragment of *OsMTP11* (LOC_Os01g62070) was amplified by high-fidelity RT-PCR based on information from the Rice Genome Annotation Project (RGAP^38^). *OsMTP11* is confirmed to consist of six exons, encoding a protein with 415 amino acids, sharing 81.3% identity with AtMTP11. Attempts to amplify *OsMTP11*.*1* (LOC_Os05g38670) full-length cDNA (based on information from RGAP) failed, despite testing RNA templates obtained from different organs (shoot, panicle and root) in different conditions (control and excess Mn and Zn). The *OsMTP11*.*1* coding sequence consists of eight exons, and is predicted to encode a protein with 418 amino acids, sharing 77.8% identity with OsMTP11, and 72% identity with AtMTP11. Sequence analysis with Phobius predicts six transmembrane domains (TMDs) for OsMTP11, and four TMDs for the OsMTP11.1 codon sequence obtained from RGAP. Both are predicted to possess cytoplasmic N- and C-termini (Fig. 1a). Alignment of OsMTP11 and OsMTP11.1 with AtMTP11 indicates that both carry the CDF signature sequence and the DxxxD domain of TMD2^19^. However, OsMTP11.1 does not possess the DxxxD domain of TMD5; this is only present in AtMTP11 and OsMTP11 (Fig. 1b).

### Analysing expression of *OsMTP11* in rice

**Figure 2:**
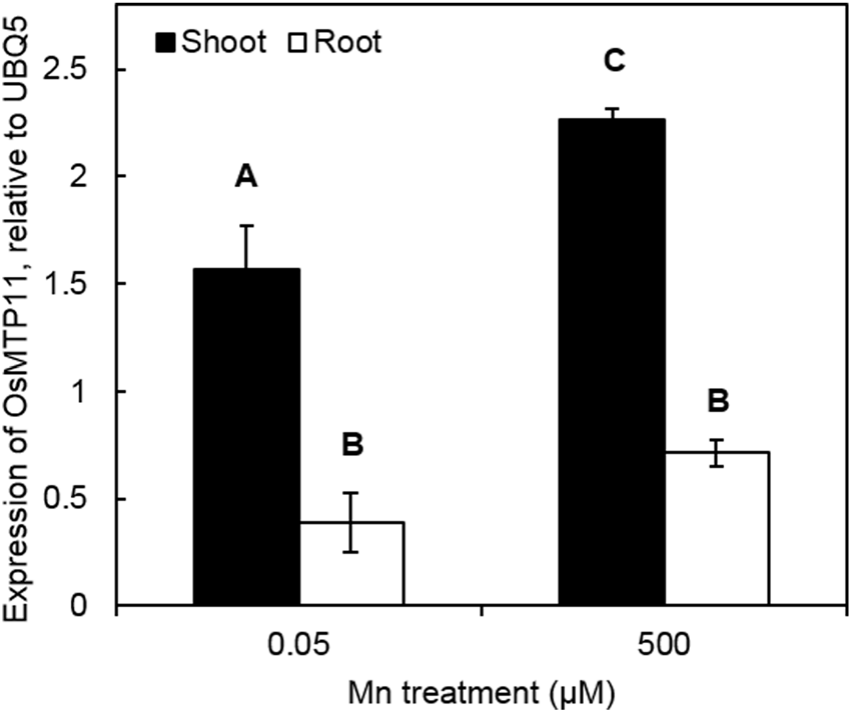
Expression analysis of *OsMTP11* in rice under Mn toxicity. *OsMTP11* expression in shoots and roots of rice plants submitted for one day to 0.05 µM of Mn (control) or 500 µM (Mn excess), evaluated by qPCR. Values are the averages of three biological replicates ± SE. Different letters indicate that means are significantly different, Tukey test (P ≤ 0.05).

The expression pattern of *OsMTP11* in rice was analysed using qPCR. Under basal Mn conditions, *OsMTP11* is expressed at higher levels in the shoot than the root. Plants were also exposed to elevated Mn conditions, at 500 µM Mn for 24 hours. This treatment induces upregulation of *OsMTP11* in shoots compared to basal conditions; no significant change was observed in roots (Fig. 2). This corresponds with findings from the Rice Oligonucleotide Array Database^39^. According to microarray data from this database, *OsMTP11* was highly expressed in shoots, particularly in leaves, with lower expression in roots, seeds and reproductive tissues. Meanwhile, *OsMTP11*.*1* had very low expression in the organs analysed with microarray, which would correspond with our lack of success in amplifying this sequence (Supplementary Fig. S1). The microarray expression profile of *OsMTP11* and *OsMTP11*.*1* across different stages of development is also shown (Supplementary Fig. S1). This data suggests *OsMTP11* is highly expressed from first leaf emergence to tillering stage.

### OsMTP11 rescues the Mn-sensitivity of the Arabidopsis *mtp11* knockout mutant

**Figure 3:**
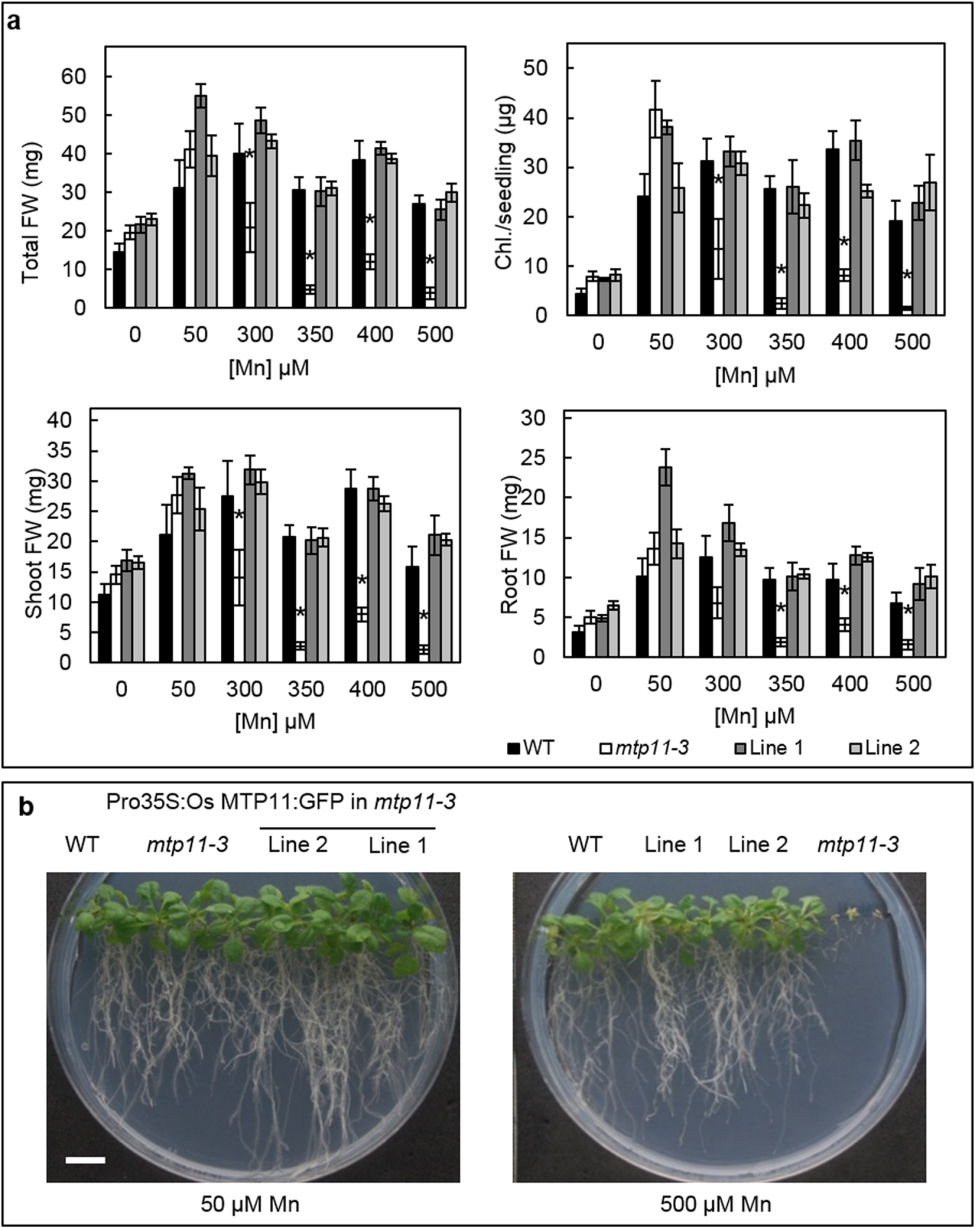
Expression of OsMTP11 rescues Mn-hypersensitivity of *mtp11-3*. Comparison of Arabidopsis Col 0 wild type (WT), *mtp11-3* and two independent Arabidopsis *mtp11-3* lines expressing Pro35S:OsMTP11:GFP when grown for 21 days on ½ MS supplied with a range of MnSO_4_ concentrations. (**a**) Data shows mean total, root and shoot fresh weight (FW; mg) and mean chlorophyll (Chl.; µg) per seedling calculated for six plates (±SE) with four seedlings per genotype per plate. Asterisk indicates that a mean of one genotype is significantly stunted compared to WT at that particular concentration, according to two-way ANOVA and Tukey post hoc test (*P ≤ 0.05). (**b**) Image displaying plant growth on different Mn concentrations. White bar = 1 cm.

We examined whether rice OsMTP11 functions in a similar way *in planta* to the Arabidopsis homolog, AtMTP11, which operates as a Mn-transporter. This was explored using a functional complementation approach whereby *OsMTP11* was expressed in the Arabidopsis *mtp11-3* mutant under the *CaMV35S* promoter. Transgenic plants were confirmed at the RNA level to be successfully expressing *OsMTP11* (Supplementary Fig. S2). The *mtp11-3* mutant has previously been shown to display reduced shoot weight and root growth rate compared to wild type (WT) above 500 µM Mn^32^, but this particular knockout mutant has not been extensively characterised. Here, we show that under control levels of Mn (50 µM Mn) and when Mn is omitted from the media (0 Mn), *mtp11-3* behaves similarly to the WT. However, fresh weight (FW) and chlorophyll levels in *mtp11-3* are significantly decreased above 300 µM Mn, when compared to control conditions and to the WT (Fig. 3). Expression of OsMTP11 clearly rescues the Mn-hypersensitive phenotype to the level of the WT, as demonstrated in the independent transgenic lines shown. Additionally, C-terminally tagging OsMTP11 with GFP does not appear to interfere with function; both tagged (Fig. 3) and non-tagged OsMTP11 (Supplementary Fig. S3) are able to restore WT-like growth on elevated Mn. This may suggest OsMTP11 serves a similar Mn detoxification function or mechanism to AtMTP11 *in planta*. *OsMTP11* was also expressed in WT Arabidopsis (Supplementary Fig. S2) and the resulting lines were tested under Mn deficiency and excess. OsMTP11 did not confer hypertolerance to elevated Mn conditions when expressed in WT Arabidopsis (Supplementary Fig. S4).

### OsMTP11 is localised to the Golgi *in planta*

**Figure 4:**
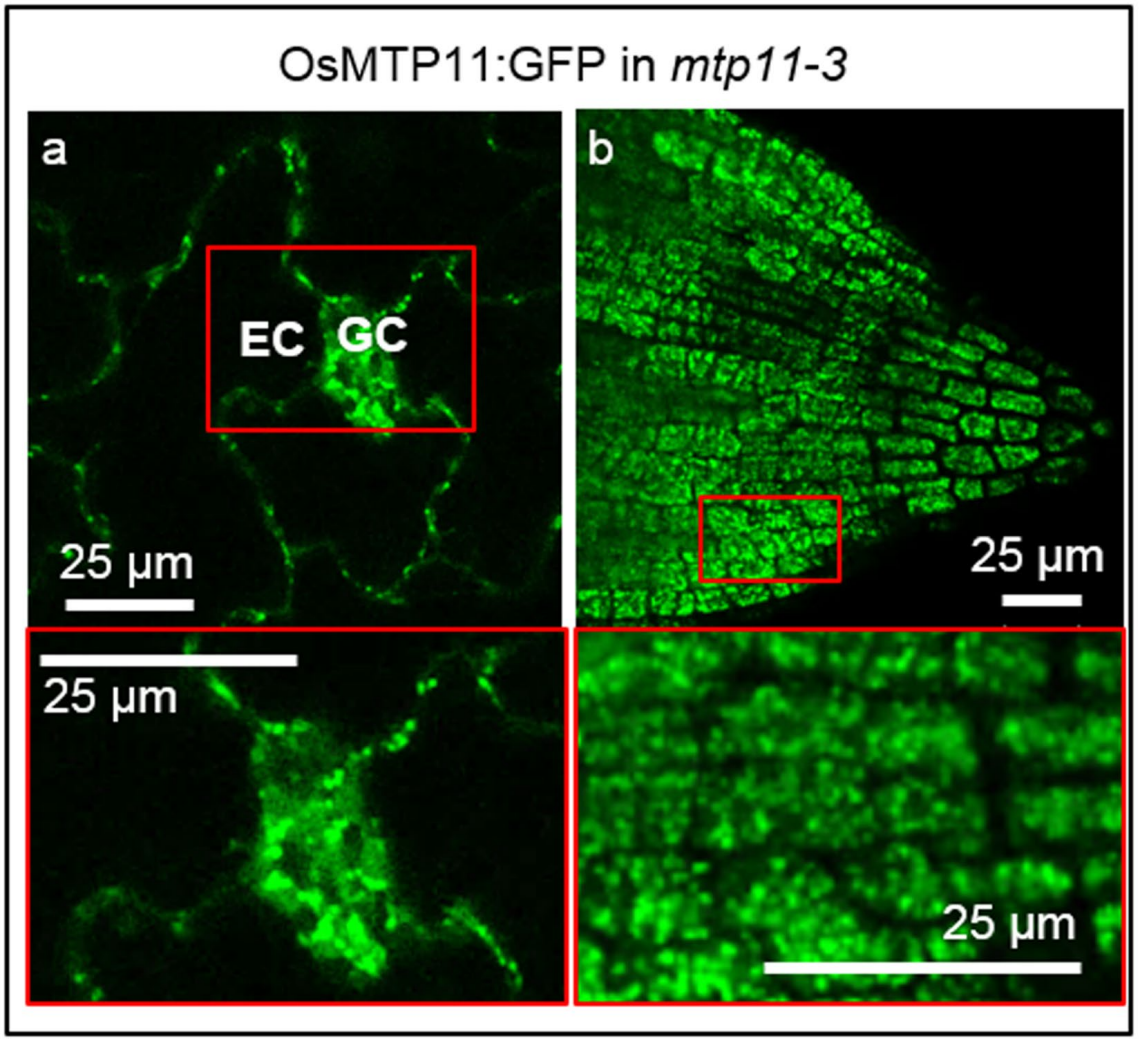
OsMTP11 localisation when expressed in Arabidopsis. Punctate expression was seen in roots and shoots when Pro35S:OsMTP11:GFP was stably expressed in 7 day old *mtp11-3 Arabidopsis* seedlings (**a**, epidermal cell, EC; guard cell, GC; **b**, root tip). Areas of higher magnification are shown in red boxes. White scale bar, as labelled.

**Figure 5:**
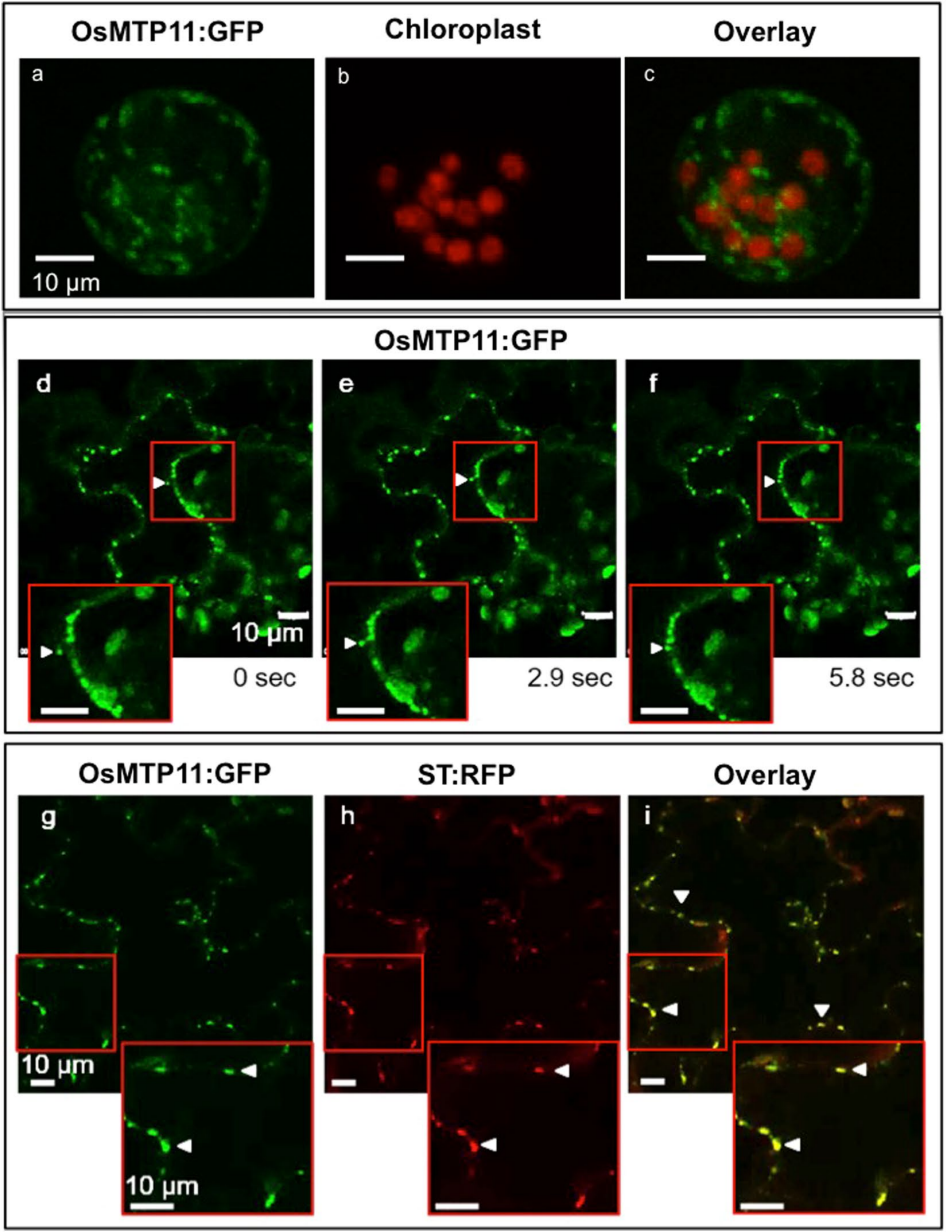
OsMTP11 localises to the Golgi. Punctate expression of Pro35S:OsMTP11:GFP when transiently expressed in rice protoplasts (**a**–**c**) and in tobacco epidermal cells (**d**–**i**). (**a**) GFP signal in rice protoplasts; (**b**) chlorophyll autofluorescence in rice chloroplasts; (**c**) overlap of a and b. (**d**–**f**) Time-lapse of OsMTP11:GFP movement around tobacco epidermal cells at 2.9 second intervals. Arrows track punctate movement around cell. (**g**–**i**) Overlap of OsMTP11:GFP signal (**g**; green signal) with TGN-marker sialyl transferase:RFP (ST:RFP; **h;** red signal) in tobacco epidermal cells. Overlap is yellow (**i**). Filled arrows, examples of punctate signal. Areas of higher magnification are shown in red boxes. White scale bar, as labelled.

To determine the subcellular localisation of OsMTP11, the Arabidopsis *mtp11-3* lines stably expressing and rescued by OsMTP11:GFP were examined under confocal fluorescence microscopy. OsMTP11 localised to small, punctate structures in both root and shoot cells (Fig. 4a,b). OsMTP11:GFP was also transiently expressed in rice protoplasts (Fig. 5a–c) and tobacco epidermal cells (Fig. 5d–i), displaying the same pattern of punctate structures. As demonstrated by time-lapse images (Fig. 5d–f) and Supplementary Movie S1, these structures were motile and reminiscent of the Golgi. Transient expression in tobacco confirmed targeting to the Golgi, with OsMTP11:GFP overlapping clearly with the Golgi co-expression marker sialyl transferase, ST:RFP (Fig. 5g–i).

### OsMTP11 functionally complements Mn-sensitive yeast mutants

**Figure 6:**
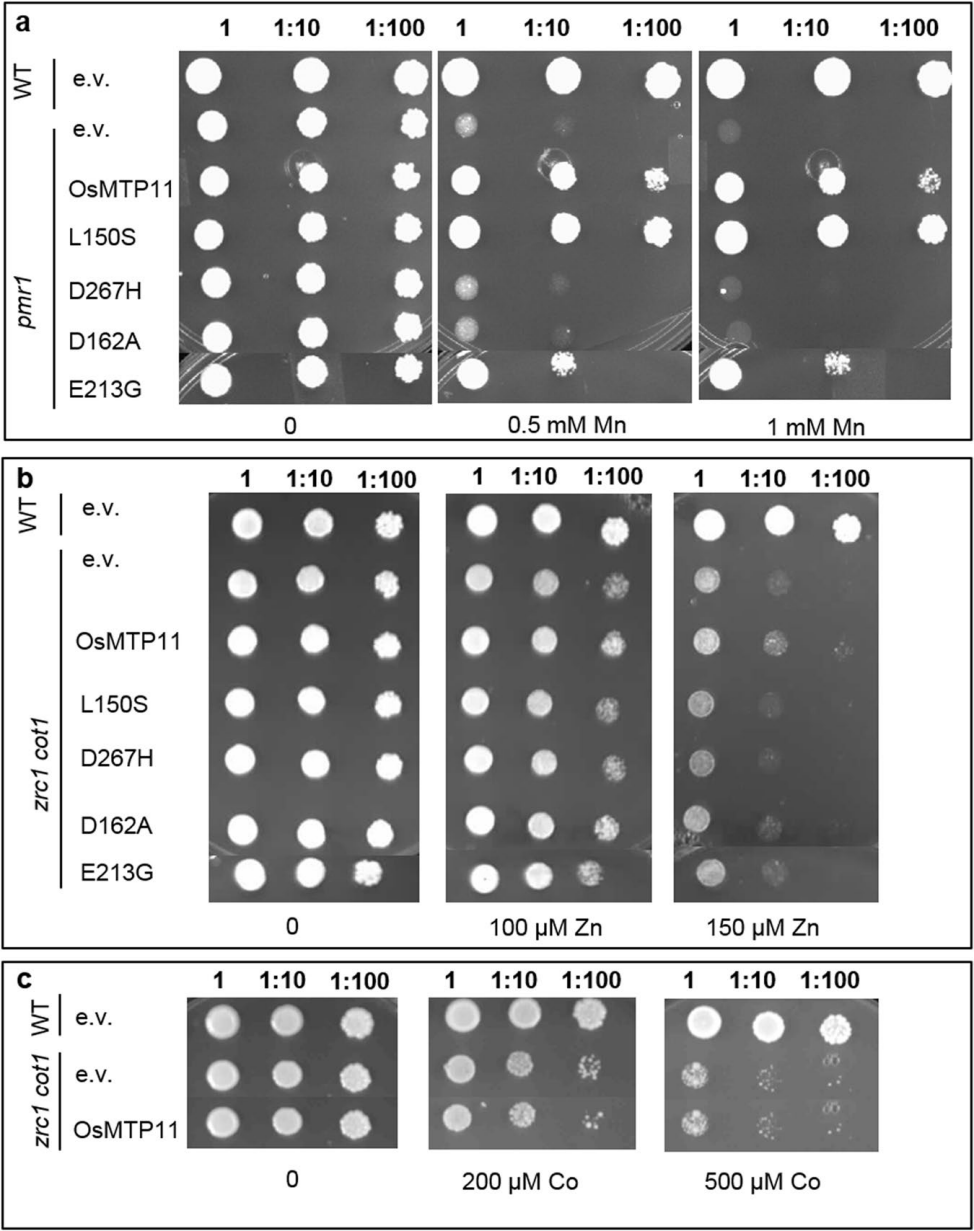
OsMTP11 rescues Mn-sensitivity of *pmr1*. Expression of OsMTP11 and four site-directed OsMTP11 mutant proteins in metal-sensitive yeast mutants: (**a**) Mn-sensitive *pmr1*, (**b**) Zn- and (**c**) Co-sensitive *zrc1cot1*. Serial dilutions of yeast cells in liquid SC galactose without uracil: undiluted (1) OD _600_ = 0.4, 1:10 and 1:100, dropped onto SC galactose without uracil (control) and supplemented with either (**a**) MnCl_2_, (**b**) ZnSO_4_ or **c**) CoCl_2_. E.V., empty pYTV vector. Plates were incubated for 5 days at 28 °C.

*PMR1* encodes a yeast secretory pathway Ca/Mn-ATPase that is located in a Golgi-like compartment^40^. Yeast *pmr1* deletion strains lack this Golgi route for Mn detoxification and are thus hypersensitive to elevated Mn^41^. The *pmr1* mutant has been used previously to support a Mn transport function for AtMTP11^32^. To further characterise OsMTP11 and investigate its specificity, *OsMTP11* was transformed into *pmr1* and other metal-sensitive yeast mutants. Expression of OsMTP11 in *pmr1* partially rescues the mutant phenotype up to 1 mM Mn (Fig. 6a). We also tested for complementation of yeast mutant *zrc1cot1*, sensitive to Zn and Co. Expression of OsMTP11 showed a very slight rescue of Zn sensitivity but did not confer Co tolerance to the *zrc1 cot1* mutant strain (Fig. 6b,c). These findings are consistent with OsMTP11 predominantly functioning in Mn transport.

### *OsMTP11* site-directed mutations

**Figure 7:**
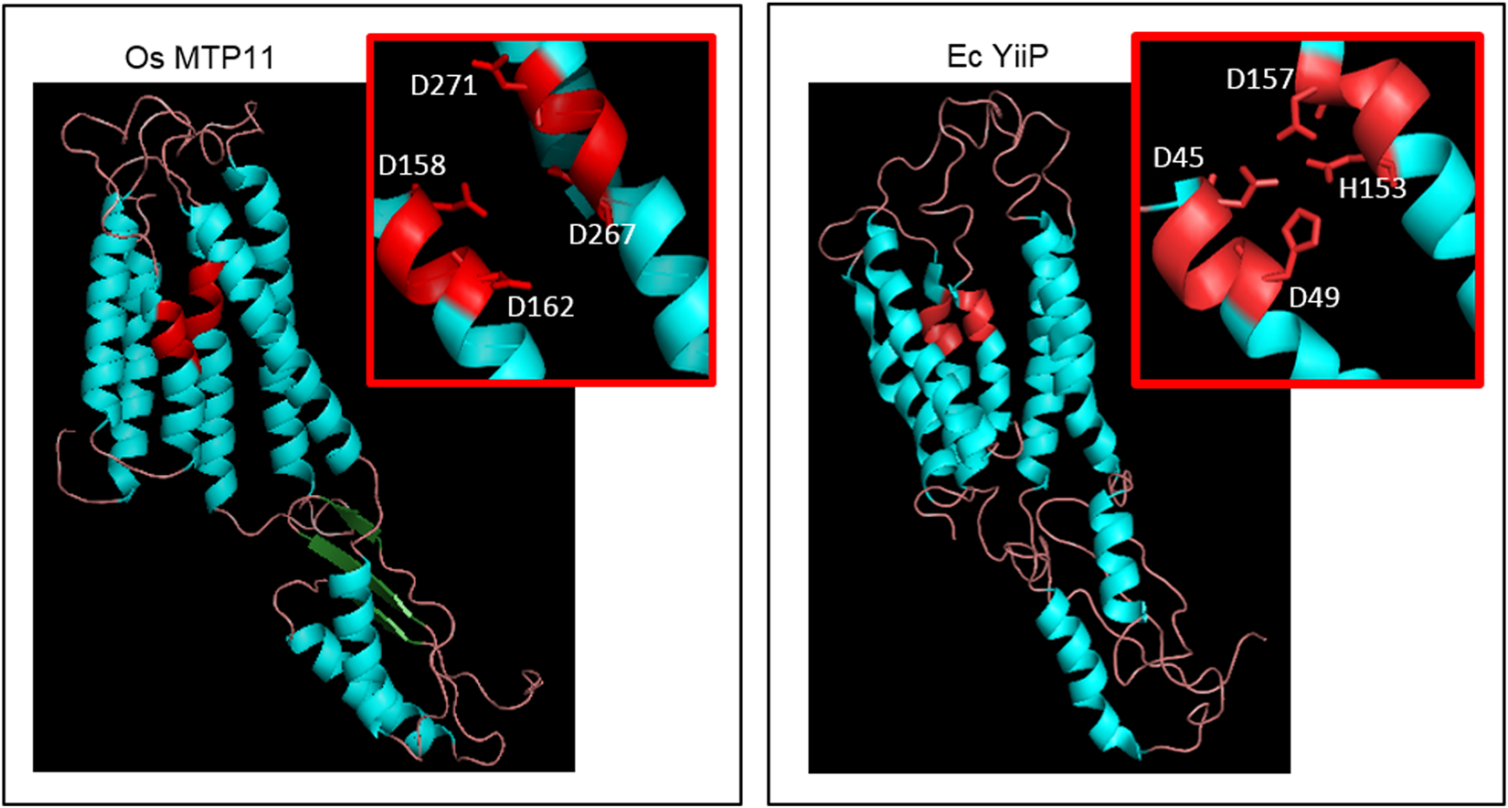
Predicted tertiary structure of OsMTP11 compared with bacterial EcYiiP. Based on homology model using Ec YiiP as template. Model constructed and visualised using Swiss-Model^70^ and The PyMOL Molecular Graphics System, Version 1.8 Schrödinger, LLC. Red inset focuses on DD-DD (OsMTP11) and DD-HD (EcYiiP) domain, which may form the ion-binding site.

Regions of conservation between MTP11 and MTP11.1 from rice and Arabidopsis are indicated in the alignment in Fig. 1. Four residues were selected for site-directed mutagenesis in OsMTP11 because they are fully conserved between these protein and may have functional significance, based on findings when equivalent residues were mutated in related proteins from other species. The selected residues are highlighted on the alignment in Fig. 1 and the predicted OsMTP11 topology diagram (Supplementary Fig. S5): L150S (TMD2), D162A (TMD2), E213G (intracytosolic loop 2) and D267H (TMD5). D162A and D267H were chosen as they fall within the conserved DxxxD domains of TMDs 2 and 5, respectively; the homologous DxxxD/HxxxD domains of the bacterial Zn-CDF EcYiiP are proposed to coordinate Zn-binding via a DD-HD coordination site^42,43^. The equivalent DxxxD domains in OsMTP11 are hypothesised to form a similar ion-binding site, coordinated by DD-DD site. This hypothetical DD-DD site is visualised in the predicted tertiary structure of OsMTP11, using EcYiiP as a homology template (Fig. 7). Thus, the D162A and D267H mutations would replace this hypothetical site with DA-DD and DD-HD residues, respectively. L150S was selected because the equivalent mutation in OsMTP1, L82S, abolishes Zn, Co and Fe transport^34^. Meanwhile, the equivalent mutation to E213G in the yeast ScZRC1, E97G, completely shifts transporter specificity^44^. L150S and E213G were thus selected to investigate whether these residues are also important for coordinating transport and specificity in OsMTP11.

The OsMTP11 mutants were expressed in sensitive yeast mutants, and their ability to restore metal-tolerance compared to non-mutated OsMTP11 was determined. Almost all mutations reduced the ability to restore Mn-tolerance in *pmr1* to some extent; this was clear in E213G, but most apparent in D267H and D162A which entirely abolished the ability to rescue Mn sensitivity. The exception was L150S, which fully rescued *pmr1* Mn-sensitivity at all concentrations tested, even conferring slightly greater tolerance to Mn than non-mutated OsMTP11 (Fig. 6a). Although non-mutated OsMTP11 showed a slight rescue of the *zrc1 cot1* Zn-sensitivity, the same was not observed in any of the four mutations tested, abolishing the slight ability to rescue Zn-sensitivity (Fig. 6b).

## Discussion

Mn is an essential micronutrient in plant nutrition, playing important structural or cofactor roles in many proteins and biochemical reactions^17^. However, Mn is required in very small quantities and toxicity symptoms such as root-cracking, chlorosis of the leaf and secondary ion deficiencies are observed under conditions of excess, potentially leading to agricultural yield losses^13,16,45,46^. CDFs from Group 8 and 9 are important for plant Mn homeostasis. Group 9 shows evidence of a duplication event prior to the monocot/eudicot split. This duplication event produced two Group 9 lineages in higher plants, both of which are maintained, and in some cases expanded^19,20,47^. Rice is usually cultivated under flooded conditions, where Mn availability is very high due to the reducing environment. Rice is a fairly Mn-tolerant species^16^ and CDFs have been shown to play an important role in this. OsMTP8.1 from Group 8 contributes to Mn tolerance by sequestration in the shoot vacuole^26^ whereas OsMTP8 is yet to be characterized. OsMTP9 from Group 9 functions mainly in the root and exists at the plasma membrane, transporting Mn into the root stele for transport to the shoot^23^.

There are two MTP11 representatives in the rice genome named OsMTP11 and OsMTP11.1 and here we focused on OsMTP11. It was not possible to amplify Os*MTP11*.*1* transcripts from different rice organs, even using plants exposed to different metal excess treatments. The expression pattern of *OsMTP11* and *OsMTP11*.*1* genes based on microarray meta-analysis clearly shows that Os*MTP11*.*1* expression is very low in most organs and developmental stages analysed (Supplementary Fig. S1). Additionally, the alignment in Fig. 1 indicates OsMTP11.1 is lacking transmembrane domain (TMD) five and thus the xxxxD domain conserved in almost all CDFs^19^. *OsMTP11*.*1* could therefore be a non-functional genomic sequence, or may have evolved as a pseudogene; however further studies are required to investigate this possibility.

Our findings suggest that OsMTP11 likely functions in Mn homeostasis to protect against moderate levels of Mn. The microarray expression profile of Os*MTP11* suggests it is mainly detected in shoots, particularly in leaves (Supplementary Fig. S1). Our qPCR data on rice tissue confirms that OsMTP11 is expressed at higher levels in shoots than roots, with increased expression in shoots when exposed to elevated Mn for 24 hours (Fig. 2).

### OsMTP11 functions as a Golgi-localised protein and contributes to Mn tolerance

Our results are consistent with OsMTP11 functioning as a Mn transporter *in planta*. AtMTP11 is a Mn transporter in Arabidopsis and when knocked out the resulting plant mutants show sensitivity to Mn toxicity^32^. The *mtp11-3* Arabidopsis mutant is characterized in more depth here. We demonstrate it has no marked growth defect compared to WT under Mn deficiency and its growth is inhibited compared to WT and control conditions only under elevated Mn, providing further support for AtMTP11 functioning to alleviate Mn toxicity in Arabidopsis. A similar role for OsMTP11 is supported by its ability to rescue the Mn-hypersensitivity of *mtp11-3*; OsMTP11 restored the growth of this mutant to WT levels when exposed to elevated Mn. A determination of the Mn content of the transformed plants would be useful to help define the underlying mechanism. Comparable complementation results were obtained for GFP-tagged (Fig. 3) and non-tagged (Supplementary Fig. S3) constructs, suggesting C-terminal tagging of OsMTP11 with GFP does not interfere with its function.

A recent study^30^ claimed targeting of OsMTP11 to the cytoplasm, plasma membrane and endomembrane system, based on bioinformatic analysis and transient expression in onion epidermal cells without markers. In contrast, we conclude OsMTP11 targets the Golgi. This conclusion is drawn from expression in three systems: OsMTP11:GFP targets a punctate, mobile organelle in both root and shoot when stably expressed in Arabidopsis or transiently expressed in rice protoplasts and tobacco epidermal cells. A Golgi-localisation was confirmed by co-expressing subcellular markers in tobacco (Fig. 5). Our observations correspond with the proposed subcellular localisation for AtMTP11 at the Golgi^32^, where it is hypothesised to function in sequestering Mn into this organelle for vesicular trafficking to the plasma membrane and eventual efflux from the cell. Similarly, the MTP11 homolog from *Beta vulgaris* spp. *maritima* is proposed to target the Golgi^48^.

### OsMTP11 partially complements the Mn-hypersensitive phenotype of the *pmr1* yeast mutant

Expression of OsMTP11 in the *pmr1* yeast mutant partially rescues the Mn-sensitive phenotype supporting a role for OsMTP11 in Mn transport (Fig. 6a). These findings suggest OsMTP11 can partially substitute for PMR1 in Mn detoxification of a Golgi-associated component in yeast, although we cannot rule out a role in cytoplasmic-binding of Mn. Interestingly, AtMTP11^32^ and OsMTP8.1^26^, which also target intracellular membranes when expressed in *pmr1*, restore its growth in the presence of 3 and 8 mM Mn, respectively, whereas growth with OsMTP11 starts to be slightly inhibited at 1 mM Mn (Fig. 6a). When we expressed OsMTP11 in WT Arabidopsis (Supplementary Fig. S4), it did not confer any additional tolerance to elevated Mn, compared to the WT. This contrasts with overexpression of AtMTP11, which confers hypertolerance to WT Arabidopsis^32^. This may imply that AtMTP11 is a more efficient transporter at elevated Mn than OsMTP11. Based on these findings, we hypothesise that OsMTP11 operates across a low-to-moderate concentration range to sequester Mn within the Golgi, and that other transporters, such as OsMTP8.1, play a greater role in Mn homeostasis at higher concentrations. It is important that rice has robust detoxification mechanisms, enabling high Mn-tolerance in its frequent reducing environment. In the future, it may also be interesting to determine whether OsMTP11 plays a role in providing physiological levels of Mn to the Golgi under deficiency conditions, or whether its function is restricted to detoxification.

OsMTP11 and AtMTP11 share 78% amino acid identity; the reason for their difference in Mn tolerance provision is not clear, but it may indicate some functional or regulatory differences. As shown in the alignment in Fig. 1, both AtMTP11 and OsMTP11 possess the MTP signature sequence and key DxxxD domains on predicted TMDs 2 and 5. The main differences in the primary sequences of AtMTP11 and OsMTP11 are found in the N-terminus before TMD1. Most Mn-CDFs possess a serine-rich N-terminus^19^; OsMTP11 and AtMTP11 possess 5 and 6 serine residues before the first TMD, respectively. The TMDs and C-terminus of the human Mn-CDF SLC30A10 have been shown to be important for coordinating Mn transport and capability^35,36^, but it may also be interesting to investigate differences in the plant Mn-MTP N-terminus, relating to function.

OsMTP11 also provided a slight rescue of *zrc1 cot1* on Zn, (Fig. 6b) but did not complement the same mutant strain for Co-sensitivity (Fig. 6c). Results presented here for yeast rescue therefore suggest that OsMTP11 is primarily associated with Mn transport but may have some affinity for Zn. The substrate specificity of OsMTP11 could therefore be broader than that of the Mn-specific AtMTP11 and PtdMTP11^32^. Interestingly, another Group 9 CDF member from cucumber, CsMTP9, is also able to transport Zn in addition to Mn^22^.

### Critical residues for the function of OsMTP11

There is little information available regarding the true structural basis of metal ion binding and coordination in Mn-CDFs. Most information so far is based on the crystal structure of the bacterial Zn-transporting CDF, EcYiiP, proposed to function as a homodimer that coordinates Zn transport at three key sites, A, B and C. Site A is coordinated by the DxxxD and HxxxD domains on TMDs 2 and 5, respectively, which are proposed to form a DD-HD coordination site^42,43^. Within the plant MTPs, most Zn transporters possess HxxxD domains on both TMDs 2 and 5, while most Mn transporters possess DxxxD domains^19^. The homology model in Fig. 7 suggests OsMTP11 may form a DD-DD coordination at site A, which may be important for function. Although the exact residues are not strictly conserved between kingdoms, site-directed mutagenesis studies have shown these domains to be important for CDF function in a range of species. For example, residues within the HxxxD/HxxxD domains of Zn-transporting OsMTP1^34^, and the NxxxD/HxxxD domains of the human Mn-CDF SLC30A10^35,36^ and rice Mn-CDF OsMTP8.1^33^ have been shown to be important for function when mutated proteins were expressed in yeast.

We therefore chose to mutate these domains in OsMTP11 to determine their importance for function, substituting the hypothetical DD-DD coordination site for DA-DD (D162A) and DD-HD (D267H). Both mutations abolish both Mn and Zn rescue ability compared to WT OsMTP11, suggesting these residues are essential for function (Fig. 6a). Equivalent mutations to D162A in Zn transporters AtMTP1^49^ and OsMTP1^34^ and Mn transporters OsMTP8.1^33^ and human SLC30A10^35,36^ also abolish transporter function in yeast. This mutation effectively substitutes the negatively-charged side chain of aspartate for the neutral alanine. Thus, the conserved aspartate of the xxxxD domain of TMD2 is essential for CDF/MTP function, either by binding the positively charged ions, such as Mn and Zn, or by maintaining the structure of the xD-xD binding site A. No increase in Zn-tolerance was conferred by the D267H mutation, which substitutes the DxxxD binding site A for the HxxxD of Zn-transporting EcYiiP. This likely indicates that other residues and domains, such as the putative site B and C, also contribute to selectivity of OsMTP11.

The L150S mutation falls in the first non-cytoplasmic loop at the beginning of the CDF signature sequence^19,47^. This mutation fully rescued the *pmr1* Mn-sensitivity, and the mutant was apparently more effective in Mn transport than the WT protein (Fig. 6a). L150S also abolished Zn transport (Fig. 6b). The leucine is equivalent to that in the L33F mutation in ScZRC1 and L82F of OsMTP1, which reduced Zn transport but increased Fe affinity, and Mn affinity in ScZRC1^34,44^. It may be concluded, therefore, that L150 is an important residue for determining metal selectivity in CDFs from different kingdoms.

E213G is homologous to E97G, a mutation in ScZRC1 that completely shifts the transported substrate from Zn to Fe and Mn^44^. Contrastingly, the corresponding AtMTP1 mutant extends its transported substrate from just Zn to include Co and Mn^49,50^. Here we found that OsMTP11-E213G continues to function in Mn rescue, although not as efficiently as the non-mutated construct, and it abolished Zn transport. E145 is a polar residue, falling within a region containing conserved polar residues thought to be involved in metal transport across the family of CDF proteins^49–52^. It may, therefore, be a further important residue for the specificity of OsMTP11. Further studies using different techniques such as OsMTP11 random-mutagenesis will be important to discover specific residues acting in Mn-CDFs activity. Identifying residues such as L150 and E213, which are important for determining selectivity of these proteins, may be useful if crops are to be developed which are more resilient to a wider range of growth conditions.

In conclusion, our study characterises the rice OsMTP11 and its involvement in Mn homeostasis. Evidence using two heterologous systems, Arabidopsis and yeast, suggests that OsMTP11 may contribute to the Mn tolerance observed in rice; further investigation in this monocot is required to confirm this. Yeast studies suggest an ability to transport Mn, with a much lower affinity for Zn. We use site-directed mutagenesis to demonstrate the importance of conserved residues in coordinating the function of OsMTP11, highlighting the importance of the DxxxD domains in Mn-MTP function. Stable expression of OsMTP11 in the Mn-hypersensitive Arabidopsi*s* mutant *mtp11-3* restores Mn tolerance to the level of the WT, indicating a conservation of function between species. Additionally, we propose that OsMTP11 may contribute to Mn transport in the rice shoot, localised to the Golgi network, based on qPCR analysis in rice and fluorescence studies when expressed in Arabidopsis, rice and tobacco. These findings contribute to our knowledge of the Mn-MTPs in rice, which is vital for understanding how these proteins contribute to Mn distribution and tolerance in rice and potentially other monocots. This information may be essential for future biotechnological progress to address the problems of food security, such as the use of monocots for biofortification or phytoremediation.

## Material and Methods

### Growth of rice plants (Nipponbare cultivar) for leaf RNA extraction and *OsMTP11* amplification

Rice seeds of the Nipponbare cultivar were germinated for four days in an incubator at 28 °C, on filter paper soaked with distilled water, and transferred to holders positioned over plastic pots with five litres of nutrient solution (16 seedlings per pot) containing 700 μM K_2_SO_4_, 100 μM KCl, 100 μM KH_2_PO_4_, 2 mM Ca(NO_3_)_2_, 500 μM MgSO_4_, 10 μM H_3_BO_3_, 0.5 μM MnSO_4_, 0.5 μM ZnSO_4_, 0.2 μM CuSO_4_, 0.01 μM (NH_4_)_6_ Mo_7_O_24_ and 100 μM Fe^3+^-EDTA, as described previously^34^. The pH of the nutrient solution was adjusted to 5.4 by addition of 0.5 M NaOH. Plants were kept at 28 °C ± 1 °C under a photoperiod of 16 h/8 h light/dark (150 μmol m^−2^ s^−1^) for 10 days. Solutions were replaced every 3 to 4 days and leaf samples were harvested for RNA extraction.

### Growth of Arabidopsis plants (WT, *mtp11-3* mutant and Os*MTP11*-transformants)

Arabidopsis plants were grown in a controlled-environment growth room as previously described^53,54^ with a day-night cycle (23 °C 16 h light, 120 µmol m^−2^ s^−1^; 18 °C 8 h dark). Soil contained equal proportions of vermiculite, Levingtons M2, and John Innes No. 2 compost (Fargro), with 0.28 g/L Imidasect insecticide (Bayer, Canada); soil was prepared as previously described^53,54^. The *mtp11-3* single mutant (GABI_366A03, described previously^32^) was obtained from the SIGnAL T-DNA collection^55^; *mtp11-3* was confirmed homozygous at the RNA level using reverse transcriptase PCR (RT-PCR) using forward primer 5′-CTGCTCGAGTTTCACGGTAAC and reverse primer 5′-AATCTGCAATCCAAGTGTTGC, which span the insertion site.

### Amplification and cloning of *OsMTP11*

The full-length sequence of *Oryza sativa OsMTP11* (LOC_Os01g62070) was found in the databases from the Rice Genome Annotation Project^38^. Total RNA from rice leaves was extracted using the Concert Plant RNA Reagent (Invitrogen, Carlsbad, CA, USA) and treated with DNaseI. First-strand cDNA synthesis was performed with oligo dT and reverse transcriptase (M-MLV, Invitrogen) using 1 μg of RNA. *OsMTP11* full-length coding sequence was amplified from cDNA with *Pfu* DNA Polymerase (Promega) using forward primer 5′-ACCCCCTGGTTCGTGGAATAATG and reverse primer 5′-CTATTTTTCATGGGACAGAGCG. In addition, reverse primer 5′- TTTTTCATGGGACAGAGCGT was used to amplify *OsMTP11* without the stop codon, referred to as non-stop (*OsMTP11*(NS)). PCR products were cloned into the entry vector pENTR/D-TOPO, using the pENTR/D-TOPO cloning kit according to manufacturer’s instructions (Invitrogen). Nucleotide fidelity of Os*MTP11* in the entry vector was confirmed by sequencing. Entry clones were recombined with destination vectors using LR Clonase II Enzyme Mix, according to manufacturer’s instructions (Thermo Fisher Scientific). For expression in yeast, entry clones were recombined with the pYTV vector^56^. For expression in plants, entry clones carrying full-length *OsMTP11* and *OsMTP11*(NS) were recombined with pMDC32 (no GFP tag) and pMDC83 (for GFP-tagging) vectors, respectively^57^. Constructs were confirmed correct and in-frame with restriction enzyme digestion and sequencing.

### Real-time PCR

For real time PCR, plants were grown as described above. Twelve-day-old seedlings were exposed to nutrient solutions containing 0.05 µM (control) or 500 µM (Mn excess) of MnSO_4_ for 1 day. The shoot and root samples were harvested for RNA extraction and cDNA synthesis as above. Quantitative RT-PCR analysis (qPCR), was carried out in an Applied Biosystems StepOne real-time cycler as previously described^58^ using OsUBQ5 (Gene Bank ID: AK061988) as a reference gene for normalization (primers are listed in supplementary data Table S1). Data were analyzed using the comparative Ct (threshold cycle) method^59^, subjected to ANOVA and means were compared by the Tukey HSD using the SPSS Base 12.0 for Windows (SPSS Inc, USA).

### Expressing OsMTP11 in *mtp11-3* mutant plants

Plant expression constructs were transformed into *Agrobacterium tumefaciens* GV3101 cells by electroporation. The floral-dip method^60^ was used to transform Arabidopsis *mtp11-3* and WT plants with addition of 100 µM acetosyringone to the culture 3 h before dipping, to induce *vir* genes. Homozygous T3 plants were isolated by screening on plates containing hygromycin and confirmed to carry Os*MTP11* using RT-PCR with forward primer 5′-CGGTGGGGAGGAGGGGGAG and reverse primer 5′-CGCCTGCCTGAGATCGAGCG.

### Metal tolerance assay in Arabidopsis plants

*Arabidopsis thaliana* seeds from WT (ecotype Columbia-0), *mtp11-3*, and *mtp11-3* carrying *OsMTP11* at the T3 generation, were surface-sterilised in 15% (v/v) bleach for 15 min and rinsed several times with sterile water. Seeds were inoculated onto plates containing 0.8% (w/v) agarose (Melford Laboratories Ltd), 1% (w/v) sucrose (VWR Chemicals), and one-half-strength Murashige and Skoog medium^61^ (Melford Laboratories Ltd) as previously described^58^. Plates were supplemented with a range of MnSO_4_ concentrations, from 0 Mn to 500 µM Mn, with 50 µM Mn treated as the control. Seeds were stratified at 4 °C for 48 h prior to transfer to a controlled-environment cabinet (22 °C, 16 h light, 110 µmol m^−2^ s^−1^; 18 °C, 8 h dark) and plates were incubated vertically for 21 days, before collection of fresh weight (FW) data, as described previously^58,62^. Chlorophyll was extracted and calculated per seedling as described previously^58,62^. Data represents FW (mg) or chlorophyll (µg) per seedling (±SE), calculated for 6 plates per condition, with 4 seedlings per genotype per plate; data was analysed with two-way ANOVA (p < 0.05).

### *OsMTP11* constructs for yeast expression and site-directed mutagenesis

Site-directed mutagenesis was performed using the QuikChange® II XL Site-Directed Mutagenesis Kit (Stratagene) according to the manufacturer’s instructions, using *OsMTP11* in the entry vector as the template. Primers are listed in supplementary data Table S1. Mutated *OsMTP11* constructs were recombined into the yeast expression vector pYTV vector and all mutations were confirmed by DNA sequencing. Constructs were transformed into *Saccharomyces cerevisiae*: WT BY4741 (MATa, *his3* ***-*** *1*, *leu2* ***-*** *0*, *met15* ***-*** *0*, *ura3* ***-*** *0*) and *zrc1 cot1* double mutant (MATa; *his3-1*, *leu2-0*, *met15-0*, *ura3-0*, zrc1::natMX cot1::kanMX4) for Zn and Co complementation analyses, and *pmr1* mutant (*MAT a; his3-1; leu2-0; met15-0; ura3-0*; pmr1::kanMX4) for Mn complementation analyses.

### Yeast transformation

Yeast transformation was performed based on the LiOAc/PEG method^63^. Transformants were selected on SC (Synthetic Complete) media without uracil (5 g L^−1^ ammonium sulphate, 1.7 g L^−1^ yeast nitrogen base, 1.92 g L^−1^ yeast synthetic drop-out media supplement without uracil; Sigma, UK) with 2% glucose (w/v) and 2% (w/v) agar (Difco technical), adjusted to pH 5.3 before addition of agar and prior to autoclaving. Plates were incubated at 30 °C for 3 days.

### Metal tolerance assays in yeast

For metal sensitivity tests, yeast cultures were grown overnight at 30 °C in liquid SC glucose without uracil. Overnight cultures were centrifuged, washed twice and suspended in liquid SC galactose medium, with 2% galactose (w/v) in place of glucose, and incubated for a further 4 hours (30 °C, 200 rpm). Yeast cultures were diluted to an OD_600_ of 0.4, and two further dilutions of 1/10 and 1/100. 7 µL of each dilution culture was inoculated onto plates containing SC galactose without uracil, supplemented with a range of metal concentrations supplied as MnCl_2,_ ZnSO_4_ or CoCl_2_. Plates were incubated at 30 °C for 4–6 days before photographing.

### Localisation studies *in planta*

Localisation experiments were carried out as previously described^54,58,64^. To image stably transformed lines, *mtp11-3* seedlings transformed with *Pro35S:OsMTP11:GFP* were grown for 7 days on 1% (w/v) agar (Sigma Aldrich) plates, supplemented with 1% (w/v) sucrose and ½-strength MS. Seedlings were mounted on microscope slides in sterile water. For transient expression in tobacco, GV3850 *Agrobacterium tumefaciens* cells were transformed with *Pro35S:OsMTP11:GFP* constructs. Positive transformants were inoculated overnight in LB containing 25 mg ml^−1^ rifampicin, 30 mg ml^−1^ gentamycin and 50 mg ml^−1^ kanamycin. Cultures were washed and suspended in 1 mL infiltration medium (50 mm MES, pH 5.6, 0.5% (w/v) glucose, 2 mm Na_3_PO_4_, 100 μm acetosyringone) and diluted to an OD_600_ of 0.5. Cultures were pressure-infiltrated into the lower epidermis of 4–6 week old wild type *Nicotiana tabacum* plants, or into those stably transformed with sialyl transferase (ST):RFP, and incubated in glasshouse conditions for a further 48 hours. Leaf discs of approx. 0.5 cm diameter were mounted on microscope slides in water. For transient expression in rice, protoplasts were isolated as described previously^65^. PEG-mediated transfections were performed with modifications^65^. For the transfection assay, 200 μL protoplast suspension (containing 2.5 × 10^6^ protoplasts/mL) was added into 10 μL of water containing 10 μg plasmid (*Pro35S:OsMTP11:GFP* construct) in a 2 mL microfuge tube and mixed gently. Next, an equal volume (210 μL) of a freshly prepared solution containing 0.4 M mannitol, 100 mM CaCl_2_, and PEG 4000 (40%, w/v) was immediately added to the plasmid/protoplast solution and mixed by inversion. The mixture was incubated at room temperature for 10 min, after which 1 mL of W5 solution (154 mM NaCl, 125 mM CaCl_2_, 5 mM KCl and 2 mM MES, adjusted to pH 5.8 with KOH) was added for PEG dilution. Transformed protoplasts were incubated in the dark for 24–48 h at 27  °C prior to imaging.

Localisation of GFP in Arabidopsis and tobacco samples were imaged using a Leica TCS SP8 Confocal laser scanning system. GFP was excited using the 488 nm line of an argon ion laser and emission was detected between 500–530 nm. Chlorophyll was excited using the 633 nm laser and emission detected between 650–700 nm. RFP was excited using the 561 nm laser and emission detected between 570–610 nm. The GFP fluorescence of rice protoplasts was observed using an Olympus FluoView 1000 Confocal laser scanning system. GFP detection as described above; chlorophyll autofluorescence detected using an LP580 filter.

### Bioinformatics analysis

OsMTP11 and OsMTP11.1 transmembrane domain (TMD) arrangement was predicted using online helix-prediction programme Phobius^66^ (http://phobius.sbc.su.se/). AtMTP11 TMDs prediction was obtained from AramTmConsens on the ARAMEMNON database^67^. The subsequent topologies were visualised according to these predictions using Protter^68^. Percentage identity was calculated using EMBOSS Matcher (EMBL-EBI)^69^. Hypothetical tertiary structures were predicted using SwissModel^70^ using EcYiiP as a homology template, and were visualised using the PyMOL Molecular Graphics System, Version 1.8 Schrödinger, LLC.

### Gene expression based on microarray meta-analysis

Spatiotemporal expression data for rice Os*MTP11* and Os*MTP11*.*1* genes were downloaded from Rice Oligonucleotide Array Database (ROAD; http://www.ricearray.org ^39^). Specific Affymetrix probes (Supplementary Table S2) for these genes were used to analyse expression data from ROAD. Only high quality arrays were used.

## Electronic supplementary material

Supplementary material

Supplemental Movie 1
