# Supplementary material for: OsMTP11 is localised at the Golgi and contributes to Mn tolerance
Source: Sci Rep. 2017 Nov 10;7:15258. doi: 10.1038/s41598-017-15324-6 (PMC5681648; doi:10.1038/s41598-017-15324-6)
Supplement: Supplementary file 1 — Supplementary material [file 41598_2017_15324_MOESM1_ESM.pdf]

OsMTP11 is localised at the Golgi and contributes to Mn tolerance

Emily C. Farthing[1#], Paloma K. Menguer[1,2#], Janette Palma Fett[2,3], Lorraine E. Williams[1*]

[1]Biological Science, University of Southampton, Life Sciences Building 85, Highfield Campus, Southampton SO17 1BJ, Hampshire, United Kingdom.

[2]Departamento de Botânica, Instituto de Biociências, Universidade Federal do Rio Grande do Sul, Av. Bento Gonçalves 9500, Porto Alegre, 91501-970, Brazil.

[3]Centro de Biotecnologia, Universidade Federal do Rio Grande do Sul, Caixa Postal 15005, Porto Alegre, 91501-970, Brazil.

[#]these authors contributed equally to the results in this paper

*corresponding author

Emily C. Farthing ef7g09@soton.ac.uk

Paloma Koprovski Menguer paloma.menguer@gmail.com

Janette Palma Fett jpfett@cbiot.ufrgs.br

*Lorraine E. Williams L.E.Williams@soton.ac.uk (corresponding author)

Tel: +44(0) 23 80594278 Fax: +44(0) 23 8059 5159

Supplementary material

Supplementary Table S1: List of primers used for real-time and cloning Os*MTP11* site-directed mutations.

Name	Sequence
OsMTP11_F	TATACTGGATTGGCGGGTGT
OsMTP11_R	ACGAGATGATCGAGATGACG
OsUBQ5_F	ACCACTTCGACCGCCACTACT
OsUBQ5_R	ACGCCTAAGCCTGCTGGTT
OsMTP11 L150S_F	GTGTATGCTTCTGTGAGAAGTGGTTCAAGTGCTATTATTGCTTCGAC
OsMTP11 L150S_R	GTCGAAGCAATAATAGCACTTGAACCACTTCTCACAGAAGCATACAC
OsMTP11 D162A_F	CGACTTTGGATTCTCTTCTTGCCTTGTTGTCAGGATTTATCTT
OsMTP11 D162A_R	AAGATAAATCCTGACAACAAGGCAAGAAGAGAATCCAAAGTCG
OsMTP11 E213G_F	CCTTCAGATCATCCTAGGATCAGTACGCTCGTTGT
OsMTP11 E213G_R	ACAACGAGCGTACTGATCCTAGGATGATCTGAAGG
OsMTP11 D267H_F	ATAGTGAAGGCTTATGCACAGCATCATTTTTTTGATGTCATCAC
OsMTP11 D267H_R	GTGATGACATCAAAAAAATGATGCTGTGCATAAGCCTTCACTAT

Supplementary Table S2: Affymetrix probes used for analysis of Os*MTP11* and Os*MTP11.1* expression

Gene ID	Main Probeset	Other Probesets	Gene Annotation
**LOC_Os01g62070**	Os.10121.1.S1_at	Os.42490.1.S1_at<br>Os.42490.1.S1_x_at	putative, expressed
**LOC_Os05g38670**	Os.10121.1.S1_at	-	putative

Supplementary Movie 1.
Punctate localisation of OsMTP11 when stably expressed in Arabidopsis.
Timelapse movie of P35S::OsMTP11::GFP (green signal) stably expressed in Arabidopsis, and imaged after 7 days growth on 1/2 MS. Movie shows mobile, punctate expression of OsMTP11 across cell, characteristic of Golgi-localisation. Interval = 1.4 seconds.

Supplementary Figure headings

Supplementary Figure 1.
***OsMTP11* and *OsMTP11.1* organ and developmental expression in rice.**
Gene expression data and rice images were obtained using the Rice Oligonucleotide Array Database [39], and based on Affymetrix-specific probes (Supplementary Table S2). All available high quality arrays on rice organ-specific expression were used. Expression level is denoted by intensity of yellow colour. Organs (a) and developing stage (b) expression are given above. In the heatmap, blue indicates a low level of expression based on microarray data; yellow indicates high expression.

Supplementary Figure 2.
RT-PCR to detect expression of *OsMTP11* in *mtp11-3* and WT Arabidopsis.
Stable expression of *Pro35S::OsMTP11::GFP* in a) *mtp11-3* and b) WT Arabidopsis (Col-0). *ACTIN2* primers amplify products of 287 base pairs (bp) from gDNA and 204 bp from cDNA, indicating all cDNA samples are of good quality with no genomic contamination. *mtp11-3* lines are confirmed knockout with *AtMTP11* primers: products of 1178bp and 529bp amplified from WT gDNA and cDNA, respectively. *OsMTP11* primers confirm presence of transgene: product of 1161 bp amplified from transgenic lines; no product amplified from untransformed WT or *mtp11-3*.

Supplementary Figure 3.
OsMTP11 expression rescues Mn-sensitive phenotype of Arabidopsis *mtp11-3* knockout mutant.
Comparison of WT, *mtp11-3* and two independent *mtp11-3* lines expressing Pro35S::OsMTP11. Plants were grown for 21 days on ½ MS supplied with a range of $MnSO_4$ concentrations. Data shows mean fresh weight (FW; mg) per seedlings calculated for six plates ($\pm$SE), with four seedlings per genotype per plate. Asterisk indicates that a mean of one genotype is significantly stunted compared to WT at that particular concentration, according to two-way ANOVA and Tukey post hoc test (*, $P \leq 0.05$). Photos display representative plant growth on basal and toxic Mn concentrations. White bar = 1 cm.

Supplementary Figure 4.
Expression of OsMTP11 in Arabidopsis WT.
Comparison of Arabidopsis WT (Col-0) and three independent WT lines expressing Pro35S::OsMTP11::GFP. Plants were grown for 21 days on ½ MS supplied with a range of $MnSO_4$ concentrations. Data shows mean fresh weight (FW; mg) per seedlings calculated for six plates ($\pm$SE), with four seedlings per genotype per plate. Genotype means at individual concentrations are not significantly different between genotypes, according to two-way ANOVA and Tukey post hoc test. Photos display representative plant growth on basal and toxic Mn concentrations. White bar = 1 cm.

Supplementary Figure 5.
Predicted membrane topology of OsMTP11.
Predicted using Phobius; visualised using Protter[68]. TMD: transmembrane domain; EL: extracytosolic loop; IL: intracytosolic loop. Amino acid residues that were mutated in this study are marked in red (L150, D162, E213 and D267).

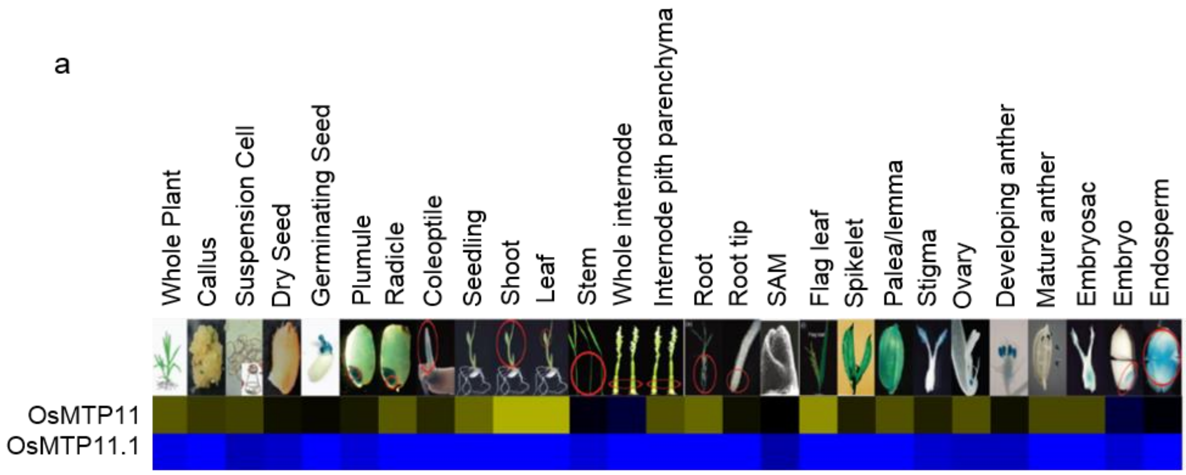

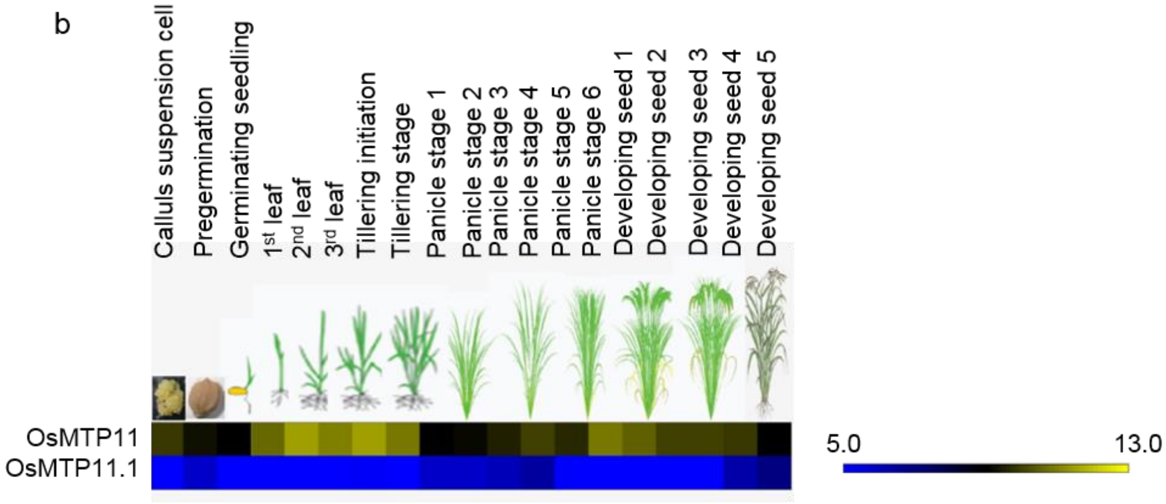

Supplemental Figure S1

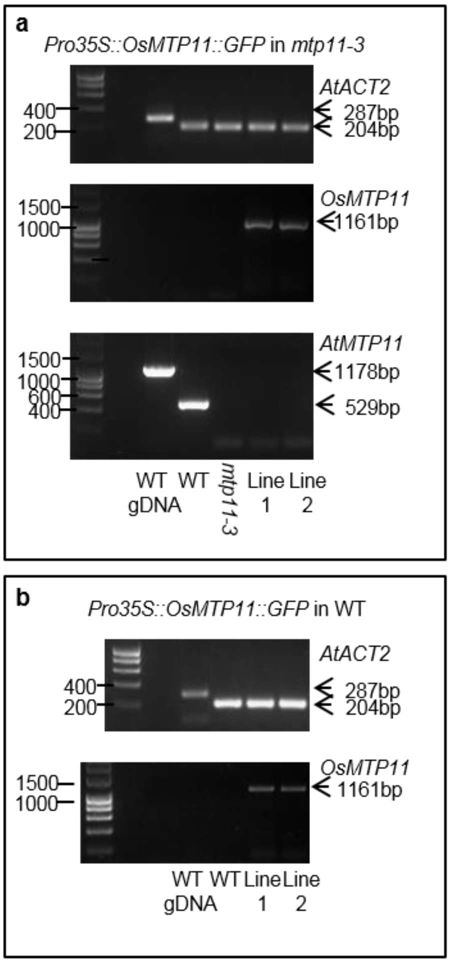

Supplemental Figure S2

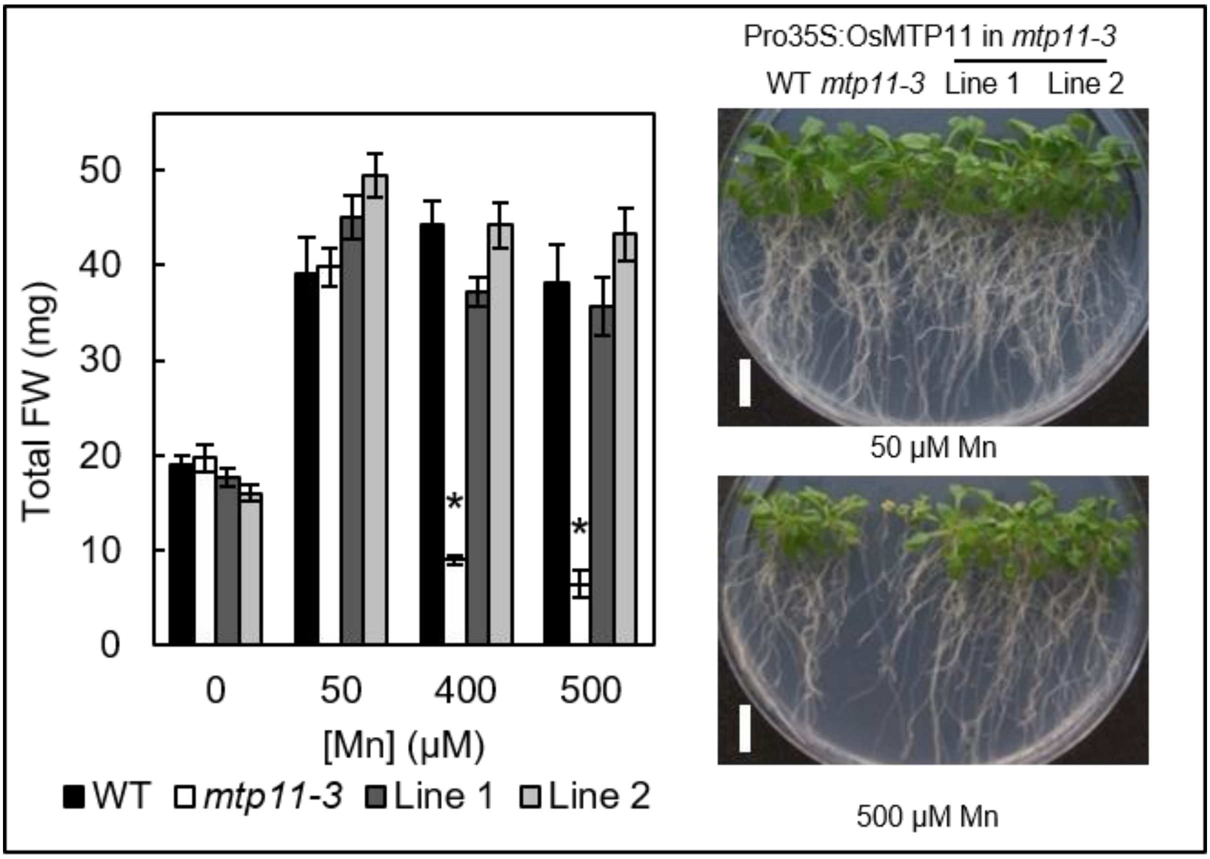

Supplemental Figure S3

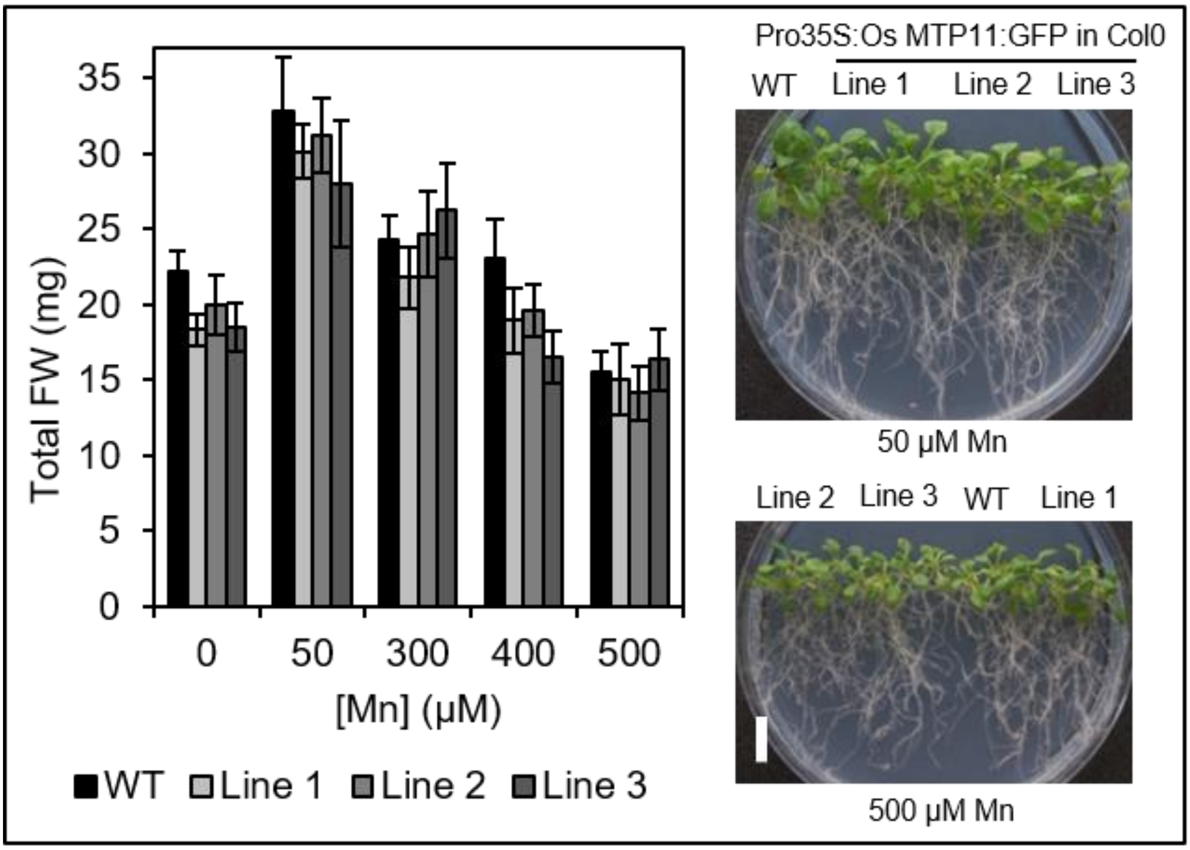

Supplemental Figure S4

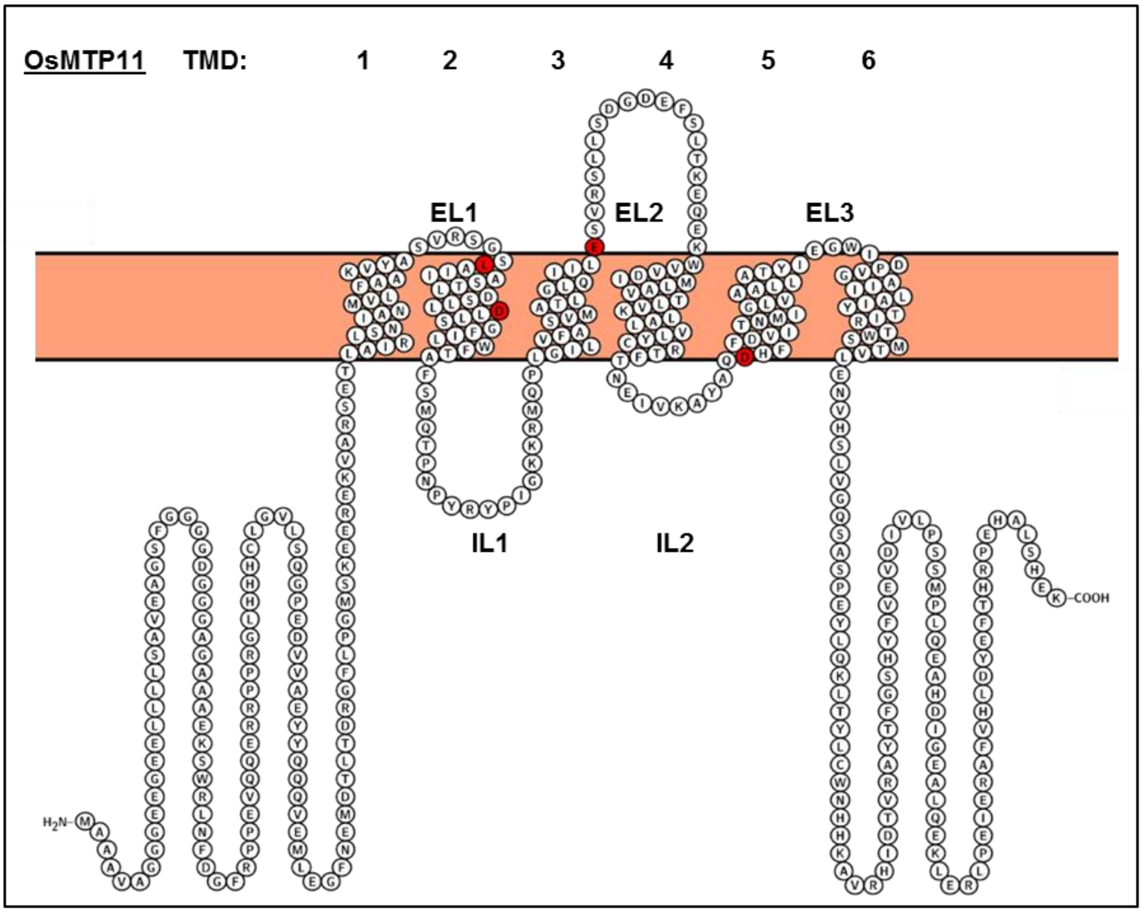

Supplemental Figure S5
